# Advances in Cardiolipin Analysis: Applications in Central Nervous System Disorders and Nutrition Interventions

**DOI:** 10.3390/biom16010071

**Published:** 2026-01-01

**Authors:** Chen Dong, Depeng Lv, Yanyan Dong, Zuohan Zhang, Quancai Li, Zhen Chen

**Affiliations:** 1 School of Pharmacy, Jiangsu University, Zhenjiang 212013, China; dongchen@stmail.ujs.edu.cn (C.D.); dongyanyan@stmail.ujs.edu.cn (Y.D.); zhangzuohan@stmail.ujs.edu.cn (Z.Z.); 2 Marine Biomedical Research Institute of Qingdao, Qingdao 266071, China; lvdepeng@ouc.edu.cn; 3 Shandong Key Laboratory of Glycoscience and Glycotherapeutics, Ocean University of China, Qingdao 266003, China

**Keywords:** phospholipids, molecular species, lipidomics, mass spectrometry, Alzheimer’s disease, Parkinson’s disease, amyotrophic lateral sclerosis, nutritional bioactive components, antioxidants, polyunsaturated fatty acids

## Abstract

Cardiolipin (CL), a unique dimeric phospholipid predominantly enriched in the inner mitochondrial membrane, is a crucial determinant of mitochondrial structure and function. Its content, fatty acyl composition, and oxidation state are associated with mitochondrial bioenergetics, dynamics, and cellular signaling. Disruptions in CL metabolism are increasingly implicated in the pathogenesis of various central nervous system (CNS) disorders, including Alzheimer’s disease, Parkinson’s disease, amyotrophic lateral sclerosis, epilepsy, and traumatic brain injury. This narrative review summarizes recent advances in the analytical techniques employed for CL analysis. The principles and applications of mass spectrometry-based platforms, nuclear magnetic resonance, Fourier-transform infrared spectroscopy, atomic force microscopy-infrared spectroscopy, and fluorescent probes were discussed, with an emphasis on their strengths in revealing the structure, composition, dynamics, and spatial distribution of CL. Furthermore, the evidence of CL abnormalities in various CNS disorders was assessed, often showing decreased CL levels, loss of polyunsaturated species, and increased oxidation associated with mitochondrial dysfunction and neuronal apoptosis. Furthermore, the nutritional interventions for CL modulation were discussed, such as polyunsaturated fatty acids, polyphenols, carotenoids, retinoids, alkaloids, and triterpenoids, which summarize their potential health-beneficial effects in remodeling the CL acyl chain, preventing oxidation, and regulating mitochondrial homeostasis. Overall, this review provided insight into integrating CL analysis and dietary modulation in understanding CL-related pathologies in CNS disorders.

## 1. Introduction

**Figure 1:**
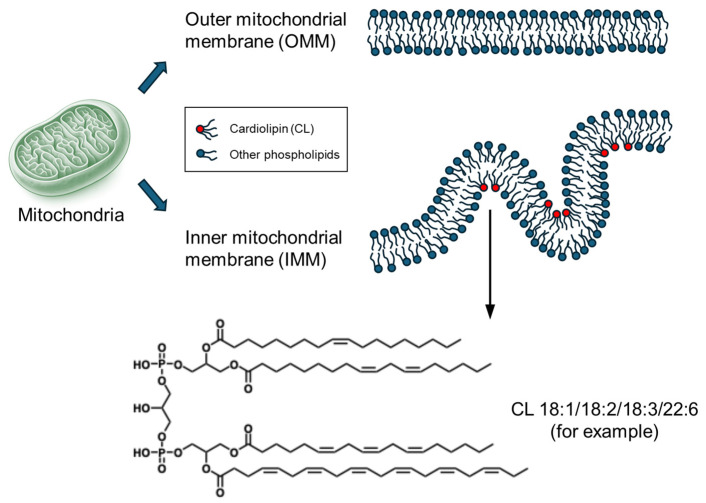
Illustrations of inner and outer mitochondrial membranes, together with the structure of CL species (using CL 18:1/18:2/18:3/22:6 as an example).

Cardiolipin (CL) is a unique tetra-acyl phospholipid exclusively localized to the inner mitochondrial membrane (IMM), accounting for 10–15% of the mitochondrial phospholipid content (Figure 1). Due to its dimeric structure and tightly packed fatty acyl chains, CL exhibits high structural stability and resistance to hydrolysis, ensuring the integrity of mitochondrial membranes and maintaining cristae architecture [1,2,3,4]. A key characteristic of cardiolipin is its distinctive negative charge distribution resulting from its two phosphate groups. The negative charge on the molecule facilitates divalent cations, such as calcium and magnesium, binding to CL. It also affects its interactions with other molecules and proteins in the mitochondrial membrane, influencing cellular signaling pathways and ion balance, while stabilizing respiratory chain supercomplexes [5,6,7,8,9]. Therefore, the complex structure and chemical characteristics of cardiolipin are vital for the organization, dynamics, and function of the mitochondrial membrane [10,11].

The concentration of CL varies among organs, among which the heart (myocardium) exhibits the highest content, reflecting its continuous and high-energy demands. Skeletal muscles, brain, liver, and kidney also contain notable amounts of cardiolipin, supporting their respective metabolic functions. In contrast, serum contains only trace amounts of CL, and its physiological role in serum is not well-characterized [12]. The crucial role of CL in mitochondrial function and brain homeostasis is further highlighted by the increasing number of neurological disorders linked with CL abnormalities [13,14,15]. In the central nervous system (CNS), nerve cells are particularly dependent on mitochondrial function. Neurons, with their extraordinary energy demands to sustain synaptic transmission, maintain ion gradients, and support complex signaling networks, are particularly vulnerable to even subtle disruptions in mitochondrial homeostasis [16,17,18,19].

**Figure 2:**
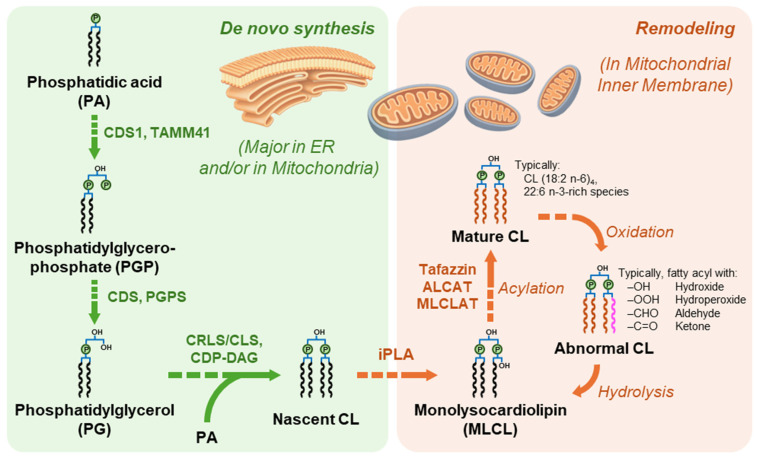
Brief pathways of CL biosynthesis and remodeling.

In mammalian cells, de novo CL biosynthesis proceeds via a phosphatidylglycerol (PG)-dependent pathway, in which phosphatidic acid (PA) is converted to CDP-diacylglycerol (CDP-DAG) by CDS1/CDS2 in the ER and/or TAMM41 in mitochondria, then to phosphatidylglycerophosphate (PGP) by PGS1, and subsequently dephosphorylated to PG by PTPMT1 [11,20,21,22]. Next, CL synthase (e.g., CRLS1/CLS1) catalyzes condensation of PG and CDP-DAG on IMM, often on the matrix-facing side [23,24]. The newly synthesized CL molecules (i.e., “nascent” CL) typically have heterogeneous, more saturated, and asymmetric acyl chains, and they are remodeled to achieve tissue-specific species with more polyunsaturated fatty acyl (PUFA) chains (i.e., “mature” CL) [24,25]. Remodeling proceeds through deacylation-reacylation/transacylation cycles, with monolysocardiolipin (MLCL) as a key intermediate [4,24]. Enzymes like iPLA2γ are implicated in CL deacylation and MLCL production, while reacylation/transacylation can be mediated by tafazzin (TAZ) [26,27,28], as well as other acyltransferases such as ALCAT1 and MLCLAT1 [29] (Figure 2). Importantly, remodeling typically enriches CL in polyunsaturated acyl chains, such as linoleate in oxidative tissues. However, the contributions of individual remodeling enzymes and the influence of enzymatic specificity versus lipid availability are context-dependent, varying across species, tissues, models, diet, and disease states. Thus, mechanistic conclusions are strongest when supported by complementary evidence from enzymology, genetics, and lipidomics [4,25,27,28]. On the other side, for CL biosynthesis, remodeling of its fatty acyls, assembly, degradation, and oxidative modification, these processes have a severe impact on mitochondrial structure and function. Under certain stress and extreme conditions, CL is externalized on the outer mitochondrial membrane (OMM) and functions as a signaling molecule to promote mitochondria fragmentation, cell mitophagy, and apoptosis [30,31,32,33].

Due to the variety of CL molecular species and complexity of their composition, it is important to clarify their nomenclature, which is primarily according to the LIPIDMAPS shorthand system [34,35]. Taking tetralinoleoyl CL (TLCL) as an example, can be annotated as: (1) CL 72:8, “lipid class (CL) + number of carbons (72) + number of double bonds (8)”, if exact mass is available and the chemical formula is decided; (2) CL 18:2_18:2_18:2_18:2, composition of every fatty acyls without position information; (3) CL 18:2/18:2/18:2/18:2, fatty acyl composition with position information. Additionally, extra information such as double bond position (e.g., 18:2 n-6), functional groups (e.g., “–OH” for hydroxy and “–OOH” for hydroperoxy), and even the proven stereochemistry (e.g., “18:2(9Z,12Z)” for the cis configuration). In terms of molecular species composition, it varies significantly across different tissues, reflecting their unique energy demands and metabolic roles. In the heart, CL is primarily composed of 18:2 n-6, with the dominant species being 72:8, featuring four 18:2 acyl chains [28,36]. This composition is maintained by enzymes such as tafazzin and ALCAT1, supporting optimal mitochondrial function and ATP production. In contrast, the brain’s CL profile incorporates a diverse array of fatty acids, particularly docosahexaenoic acid (DHA, 22:6 n-3), which is vital for neuronal function [36]. This diversity arises from the brain’s specialized lipid metabolism. The liver’s CL consists mainly of mono- and di-unsaturated chains with 16 or 18 carbon atoms [37], differing from the linoleate-rich profile of the heart, reflecting its role in lipid metabolism and detoxification. Skeletal muscle mitochondria also exhibit a C18:2 n-6-enriched CL composition, particularly in slow-twitch fibers, which is crucial for supporting oxidative phosphorylation [38,39]. While data on kidney CL is sparse, existing research suggests tissue-specific fatty acyl compositions, warranting further study to understand their functional implications. Even within the same organ, the specific fatty acid composition of CL can vary, for example, as seen in the atrium/ventricle and renal medulla/cortex [12].

CL content, acyl chain remodeling, and oxidative modifications implicate disturbances in CL metabolism, and thus, impair mitochondrial integrity, as recurring features in the diseased brain [17,18,40,41]. Therefore, it is important to clarify not only the cardiolipin content but also its composition of molecular species. Mass spectrometry (MS) is an ideal technology for qualifying cardiolipin species and quantifying the content of each species, especially fragmentation of the fatty acyls and the remaining residues by tandem MS. This approach is particularly useful in investigating the molecular chemical changes, such as oxidation, hydrolysis, degradation of intact CL, and formation of its related products. Besides the chemical structure, the unique molecular configuration of CL enables it to form non-bilayer structures under certain physiological conditions, such as membrane curvature and lipid packing defects, further expanding its functional repertoire within the cell. Hence, a comprehensive strategy composed of different methods (including alternative methods) is to conduct: nuclear magnetic resonance (NMR) and Fourier-transformed infrared spectroscopy (FTIR) for phase behavior, atomic force microscopy (AFM) for identifying CL-protein aggregates, and fluorescent probing for tracking CL translocation and distribution.

Therefore, this narrative review aims to CL analyses from different aspects and their practical applications in CNS diseases, together with nutritional supplements for CL regulation.

## 2. Strategies and Methods of Cardiolipin Analysis

### 2.1. MS-Based Platforms

The low amounts and structural diversity of CL species make them hard to analyze comprehensively. Nowadays, MS has emerged as the indispensable tool for CL profiling, capable of resolving complex species, isomers, and related products (such as lysoCL and ox-CL) in biological matrices.

#### 2.1.1. Universal Considerations in MS-Based CL Analysis

The high degree of unsaturation in mature CL species makes them highly susceptible to oxidation, which produces artifact oxidation products that interfere with the analysis of authentic ox-CL [42]. Therefore, sample storage (usually at −80 °C) and pretreatment (typically with antioxidants like BHT) require careful handling [43]. As CL exists exclusively in mitochondria, a prior extraction of cellular mitochondria followed by CL extraction and purification (e.g., solid-phase extraction) is sometimes performed.

For detection, electrospray ionization (ESI) in negative ion mode is the cornerstone of CL analysis due to the molecule’s two phosphodiester groups [44,45,46]. CL typically forms a doubly deprotonated [M−2H]^2−^ ion, which is more abundant and stable than the singly charged [M−H]^−^ ion in some reports [45,46]. The presence of alkali metals (e.g., Na^+^, K^+^) in the matrix can lead to the formation of mixed adducts (e.g., [M-2H+Na]^−^ and [M-3H+Na]^2−^), which complicate the spectra and reduce sensitivity. Positive ion mode is not commonly used in CL analysis, in which [M+NH_4_]^+^ is the characterized ion [47]. In addition, Hsu et al. reported that [M−2H+3Na]^+^ ions could be observed with low sensitivity [48]. Regarding the MS analyzer, high-resolution mass spectrometry (HR-MS) is essential because of the high prevalence of isobaric and isomeric species. Platforms like Orbitrap and FT-ICR MS allow the sub-ppm mass accuracy required to distinguish species with minute mass defects (e.g., differing in double bond equivalents or oxidation states). The combination of HR-MS and ion mobility spectrometry (IMS) in a hybrid instrument provides an additional separation dimension based on the ion’s collision cross-section, which helps resolve isomers and improve CL annotation in complex samples.

The interpretation of CL tandem MS (MS/MS) spectra is quite complex. Low-energy collision-induced dissociation (CID) of the predominant [M−2H]^2−^ ion primarily yields carboxylate anions (RCOO^−^), allowing straightforward identification of the constituent fatty acyl chains. In contrast, fragmentation of the [M−H]^−^ ion provides more information on the headgroup and phosphatidylglycerol moieties, producing fragments such as deprotonated phosphatidic acid and phosphatidylglycerol [44,46,48,49]. However, the natural occurrence of CL as a mixture of positional isomers means that the observed spectrum is often a mixture, complicating precise structural assignment without additional separation or advanced fragmentation techniques [46,50]. For the annotation and identification of the MS signals, He et al. provided an approach of building an in silico spectral library for CL, lysoCL, and ox-CL species [47].

#### 2.1.2. LC-MS

The primary advantage of LC-MS is the reduction in ion suppression and matrix effects, leading to more accurate identification and quantification, particularly for low-abundance species in complex biological matrices, such as brain tissue. Reverse-phase liquid chromatography (RPLC), particularly when using ultra-performance liquid chromatography (UPLC), has become the workhorse for resolving individual CL molecular species (including their complex metabolites and reaction products) prior to MS detection [12,51,52,53,54]. Some advanced modifications even improved LC-MS-based CL profiling, such as using 1-butanol as the RPLC mobile phase to enhance the resolution of highly hydrophobic CL species [55], performing two-dimensional separation to achieve a more comprehensive detection of CL [56,57]. Alternatively, hydrophilic interaction liquid chromatography (HILIC) can separate CL classes (as well as ox-CL) based on headgroup polarity [56,58].

#### 2.1.3. Shotgun Lipidomics

This high-throughput approach involves the direct infusion of crude lipid extracts into the mass spectrometer, bypassing chromatographic separation. Its speed makes it ideal for large-scale screening studies, such as profiling CL alterations across hundreds of samples in disease cohorts. The trade-off is the increased potential for isobaric overlap and ion suppression. Therefore, shotgun lipidomics for CL relies heavily on HR-MS and advanced data processing pipelines (e.g., LipidXplorer) to deconvolute complex spectra and ensure accurate annotation without chromatographic resolution [59,60,61,62].

#### 2.1.4. MS Imaging

MS imaging technique has substantially advanced researchers’ understanding of CL biology by preserving its spatial distribution within tissues. These ambient ionization methods enable the direct analysis and mapping of CLs on tissue sections. This has been pivotal in correlating specific CL profiles, even for ox-CL species (e.g., CL 18:2/18:2/18:2/9:1-OOH) [63], with distinct pathological regions. For instance, CL depletion can be mapped to necrotic zones in glioblastoma, and hydroperoxidized CL molecules can be localized to the mitochondrial-rich regions of tumors. Matrix-assisted laser desorption ionization (MALDI) or desorption electrospray ionization (DESI) coupled to MS imaging is applied to rapidly profile CL fingerprints in complex biological samples, like leukocytes for Barth syndrome diagnosis (detection of MLCL/CL ratio abnormalities with ~95% specificity) [64] and murine retinal cell layers for retinal pathology development and progression (comparison of PUFA in CL) [65]. In addition, coupling MS imaging with ion mobility further enhances specificity by reducing chemical noise, providing researchers a closer glance at single-cell CL profiling [66].

Nevertheless, several practical factors affect the robustness and interpretation of CL profiles on MS-based platforms. One realistic problem is that the high level of unsaturation in mature CL species makes their modifications sensitive to the handling, extraction, and storage conditions of the samples. Taking precautions may help prevent artificial oxidation or degradation, ensuring the biological interpretation remains clear and accurate [42]. Another issue is that in negative-ion ESI, salt-related adduct heterogeneity and mixed adducts can complicate precursor assignment and reduce sensitivity, particularly when the matrix composition varies [44,46,48,49]. Moreover, due to a series of instrumental conditions and parameters, cross-study comparisons are not easy to perform unless the experimental transparency (e.g., acquisition settings, ion/adduct forms, advanced MS fragmentation parameters, annotation rules, calibration/standardization methods, etc.) is sufficient.

### 2.2. Nuclear Magnetic Resonance (NMR)

NMR is a powerful spectroscopic tool that reveals molecular structures and dynamics in biomembrane studies [67,68]. It even shows static conformations and interactions of lipids and proteins [67,69], exhibiting unique advantages in characterizing the polymorphic phase behavior of CL. Due to its dimeric structure, two PG units on a single glycerol backbone with four acyl chains, CL possesses a small headgroup relative to its large hydrophobic volume, resulting in a conical shape that predisposes it to non-lamellar phases [70,71]. Regarding CL in aqueous dispersions, Vasilenko et al. studied the structure and phase behavior of CL derived from mammalian (bovine heart) and microorganism (*B. subtilis*) sources. The results indicated that sodium salts of CL primarily form bilayers at low ionic strength and neutral pH; however, when Ca^2+^, Mg^2+^, or Ba^2+^ salts are present, these bilayers transition to hexagonal phases, of which the phase changes are identified through characteristic ^31^P-NMR spectral signals [71]. Further studies examined how phase behavior varies with different solid concentrations of CL and its related metabolic products, including MLCL, di-lysoCL, and acyl CL. The findings revealed that CL shifts from a lamellar to an inverted hexagonal phase; di-lysoCL dispersions behave as micelles at low concentrations and then transition to a lamellar phase; meanwhile, MLCL remains in a lamellar phase, and acyl CL stays in an inverted hexagonal phase [70]. The different “shapes” indicate lipid polymorphism, and these results suggest the importance of head-group interactions in determining lipid phase behavior [70].

Additionally, NMR is crucial for investigating lipid-protein interactions and determining CL-binding sites. While solid-state NMR provides information on membrane-bound states, solution NMR is suitable for molecular analysis [68]. The commonly used ^1^H-NMR and ^13^C-NMR have been employed to clarify the chemical structure of CL, for instance, detecting free radical-mediated cis-to-trans isomerization in the fatty acyl chains of CL, as reported by Vetica et al. [42]. While the trans isomers exhibit a tighter packing of the lipid layer, resulting in vesicle size decrease, liposome diameter reduction, and mitochondrial membrane damage, the isomerization process can be monitored through the regions 2.0–2.8 ppm of the ^1^H-NMR spectra [42]. These results demonstrated that CL co-purifies and tightly associates with the c-subunits of ATP synthase, implying that such lipid–protein interactions are essential for enzyme complex stability [68].

### 2.3. Fourier Transformed Infrared Spectroscopy (FTIR)

FTIR demonstrates unique advantages in studying the polymorphic phase behavior of CL. Similarly to solid-state NMR, FTIR directly analyzes the intrinsic vibrations of functional groups, making it especially valuable for studying native lipid systems under physiological or near-physiological conditions [72]. By using FTIR, CL is reported to be a hexagonal crystal lattice structure in both anhydrous and aqueous samples [73,74]. Borchman et al. found that the serum protein β2-GP I interacting with CL resulted in CL conformational alteration, which could be observed through the broadening of the CH stretching bands in the FTIR spectra [75].

Monitoring multi-component membrane systems, Klaiss-Luna et al. found an interesting system in the *S. aureus* model membrane. The elevated concentrations of CL affected the systematic fluidity, increasing the transition temperature (T_m_) of the model membranes at a fixed temperature, providing evidence that CL imposes structural consequences on the bilayer core of the *S. aureus* membrane [72]. Furthermore, studies have suggested that CL elevation in *S. aureus* contributed to the modification of membrane composition, which helped enhance its resistance to antibiotics (like daptomycin) [76,77,78].

### 2.4. Atomic Force Microscopy–Infrared Spectroscopy (AFM-IR)

AFM-IR can identify the chemical structure of individual protein aggregates [79]. CL is present in insulin oligomers grown with these phospholipids [80]. Notably, saturated fatty acyls in CL reduced insulin aggregation and caused less cell toxicity than those with unsaturated fatty acyls [81]. Furthermore, Matveyenka et al. compared various lipid-insulin aggregates. They discovered that CL accelerated fibril formation, promoted insulin aggregation, and resulted in shorter fibrils compared to those formed in the lipid-free environment [82]. These results showed lipid structure affects insulin aggregate toxicity and reduces mitochondrial dysfunction.

Besides insulin, the amyloid-β (Aβ) protein was also investigated for the secondary structure and fibril formation at the early and late stages of aggregation: anionic CL showed the most substantial acceleration of Aβ aggregation, increased parallel β-sheet content in Aβ oligomers and fibrils, directly correlating with enhanced toxicity to neuronal cells [83]. According to Matveyenka et al., CL, ceramide (Cer), and sphingomyelin (SM) significantly altered the secondary structure of lysozyme aggregates, promoting β-sheet formation and reducing oligomer flexibility, which were correlated with the reduced cellular ROS production and mitochondrial dysfunction [84]. The series work using AFM-IR suggested that membrane lipid composition, including CL, could critically influence amyloid pathology [85].

### 2.5. Fluorescent Probe

Fluorescent probes are a popular and highly sensitive method for visualization, extensively used in physiological, pathological, biological, and clinical research. They work by strongly binding to CL and producing fluorescence signals that help analyze the distribution, content, and dynamics of CL within biological systems [54,86,87,88]. The most common fluorescent dye is 10-N-nonyl acridine orange (NAO), first developed in the 1980s, and has been widely used to visualize CL and mitochondria in various models of neurodegeneration and oxidative stress. Ryan et al. utilized NAO staining to assess CL exposure on the OMM for exploring α-synuclein pathology in Parkinson’s disease [89]. Qi et al. used NAO fluorescence to measure mitochondrial CL levels and identified ginsenoside Rg3 as a bioactive component acting as an agonist for GRB2 and TRKA, influencing the TRKA-GRB2-EVI1-CRLS pathway [90]. Silva et al. employed NAO to detect externalized CL in cortical neurons subjected to microbial β-*N*-methylamino-l-alanine (BMAA) stress, offering insights into mitochondrial-driven inflammatory pathways in Alzheimer’s disease [91]. Additionally, in a study on tau-induced mitochondrial membrane disruption by Camilleri et al., NAO imaging was utilized to quantify CL and demonstrated that tau proteins have a high affinity for CL-rich membranes [92].

Additionally, researchers are developing small-molecule dyes other than NAO that specifically integrate into mitochondrial membranes to visualize and measure CL. They are also exploring alternative organic compounds that can pass through membranes containing CL. Mohr et al. designed small organic fluorophores through a data-driven approach, achieving improved selectivity for cardiolipin over structurally similar phosphatidylglycerol (PG), demonstrating that tailored physicochemical properties can significantly enhance probe performance [93]. Leung et al. described TTAPE-Me as a more effective sensor for CL quantification, addressing the previous limitations in selectivity and sensitivity observed with NAO [94]. Currently, fluorometric CL assay kits are commercially available, with some featuring proprietary probes that fluoresce only upon binding to CL. These probes do not react with other lipids, such as phosphatidylcholine or sphingomyelin, ensuring high specificity. With their molecular specificity, spatiotemporal resolution, and suitability for live-cell studies, this approach has become a valuable tool for understanding CL dynamics in mitochondrial physiology and pathology. Compared to NMR or MS, fluorescence approaches provide real-time imaging, high sensitivity to low CL levels, independence from instruments, and enable dynamic tracking in live cells or isolated mitochondria [90,95].

As there are increasingly developments in MS, such as LC-MS, tandem MS, ion mobility MS, HR-MS, MS imaging, and others, researchers are now able to precisely elucidate the structural elucidation and spatial mapping of CL variants, even at very low concentrations [49,55,59,64,96,97,98,99,100,101]. NMR and FTIR spectroscopy have further enriched our approach, enabling researchers to probe CL dynamics and molecular interactions in unprecedented detail [68,69,70,71,73,74,102]. At the same time, the fluorescent probe enables a more visualized observation of CL dynamics. Another powerful tool, transmission electron microscopy (TEM), provides direct visualization of mitochondrial ultrastructure, including capillary loops and cristae conditions, along with measurements of length, width, area, and circularity, as well as interactions between mitochondria and the endoplasmic reticulum (ER) [103]. Therefore, the selection of the analytical strategy must be driven by specific research questions. Here, we have summarized a strategic guide for this selection (Table 1), summarizing how each methodological platform addresses key dimensions of CL analysis and highlighting its key features in building a comprehensive understanding of CL in biomedical applications. It should also be noted that these methods, combined with molecular biology assays like Western blots and RT-PCR for enzymes and gene expression, help with CL profiling and offer a comprehensive view of mitochondrial health.

## 3. Applications of Cardiolipin Profiling in CNS Diseases

**Figure 3:**
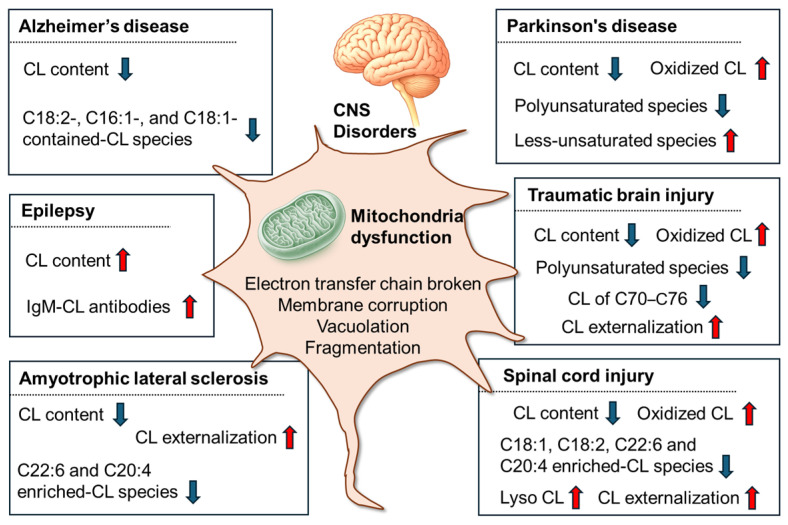
CL changes in CNS diseases associated with mitochondrial dysfunction.

CNS diseases, encompassing both neurodegenerative diseases and traumatic injuries, represent a substantial and growing global health burden. CL abnormalities have been discovered in a range of CNS disorders (Figure 3), including alterations in CL content, composition (of molecular species), degradation, and oxidation, which reveal its close association with mitochondrial dysfunction.

### 3.1. Common and Specific Characteristics of CL Signatures Across CNS Disorders

Across diverse models and tissues, many CNS disorders exhibit mitochondrial stress: reduced total CL, loss of PUFA-enriched species, and increased oxidative remodeling (i.e., driven by abnormal oxidation of CL). In Alzheimer’s disease (AD) models, several studies report a decrease in total CL and reductions in C18:2-enriched and unsaturated species, indicating a loss of “energy-competent” IMM lipids [41,104]. In amyotrophic lateral sclerosis (ALS), LC-MS lipidomics reveals a greater than 25% total CL reduction and a preferential loss of long-chain PUFA species (e.g., 22:6, 20:4) [105,106,107]. In acute injury (TBI), spatial lipid mapping reveals an early, region-specific loss of PUFA-rich CL (C70–C76, declining by ~50% in vulnerable regions) and redistribution of IMM → OMM, marking the progression from metabolic stress to mitochondrial failure [40,108]. These common trends are consistent with evidence that oxidative imbalance drives early CL peroxidation when ROS triggers antioxidant defenses [32,109,110]. Uncontrolled CL oxidation impairs oxidative phosphorylation (e.g., complex I and coupled respiration) and promotes cytochrome c release by outer membrane permeabilization. Oxidized CL can be hydrolyzed to lysoCL and oxidized fatty acids (e.g., via iPLA2γ), underlying “ox-CL/lysoCL signatures” in neuronal injury [26,32,110,111].

The same axes reveal disease- and context-specific CL signatures. In Parkinson’s disease (PD) models, reduced CL levels are observed in toxin/genetic systems, and early CL movement to the OMM occurs before fragmentation. CL-rich membranes also modulate α-synuclein aggregation, features linked to synucleinopathy biology beyond general mitochondrial stress [82,112]. Human substantia nigra lipidomics highlights cohort differences: in male PD, phospholipids decrease while CL mass may remain constant, unlike in females. Thus, “total CL” may not capture PD pathology without stratification [89,113]. In epilepsy, regionally resolved lipidomics reveal hippocampal CL enrichment compared to the cortex, interpreted as cortical oxidative loss and compensatory hippocampal synthesis during seizures [114]. They underscore that “disease specificity” can be expressed as brain-region specificity, stage dependence, and sex/genotype dependence, rather than as a single invariant lipid marker.

These CL alterations are not just biomarkers of mitochondrial disorder-involved CNS disorders but also recognized as contributing to disease pathogenesis through the following proposed mechanisms: Primarily, loss of mature CL (typically, CL (18:2)_4_) impairs respiratory chain structures, leading to inefficient ATP production, electron leakage, and ROS that causes CL oxidation. The oxidized CL externalizes on the OMM signals for cell death via mitochondrial permeabilization, releasing cytochrome c and activating caspases, which cause neuronal apoptosis common in neurodegeneration. Furthermore, ox-CL and lyso-CL act as damage-associated molecular patterns, activating microglia, triggering neuroinflammation, and worsening tissue injury. Overall, the CL signature can be used to indicate CNS disorders that reflect the specific point(s) at which this central lipid has been perturbed, initiating or amplifying one or more of these deleterious pathways that culminate in neuronal dysfunction and loss. The detailed changes in the specific CNS disease are discussed below.

### 3.2. AD

AD, pathologically characterized by extracellular Aβ plaques and intracellular tau neurofibrillary tangles [115,116,117,118], has been increasingly linked to mitochondrial dysfunction and associated lipid abnormalities [18,119,120,121]. Given the pivotal role of CL in sustaining mitochondrial homeostasis, a range of advanced analytical techniques have been applied to assess CL alterations in AD, revealing distinct molecular signatures that will be detailed in the following section.

Using shotgun lipidomics mass spectrometry, numerous studies have observed a significant reduction in cardiolipin content in SH-SY5Y cells of the AD model [122,123], accompanied by increased levels of triacylglycerols and phospholipids, which indicates oxidative stress-related lipid remodeling [122]. Similar findings have also been reported in cortical organoids and the brains of AD model mice [41,104], particularly a reduction in C18:2-enriched CL species and in those containing C16:1 or C18:1 acyl chains (e.g., typically, CL 72:8, CL 72:7, CL 72:6, CL 70:7, CL 70:6, and CL 68:6) [41]. In addition, NAO fluorescent staining to visualize CL exposure on the outer mitochondrial membrane of primary cortical neurons treated with the bacterial neurotoxin BMAA, indicating mitochondrial membrane disruption and potential mitophagy or inflammasome activation [91].

### 3.3. PD

Across patient-derived systems and PD models, mitochondrial defects closely relate to CL biology, making CL metrics useful indicators of dopaminergic neuron health. Lipidomics consistently shows decreased CL levels in toxin and genetic models, such as the 6-OHDA-injured rat cortex, A53T-αSyn-treated SH-SY5Y cells, MPTP-treated mouse substantia nigra, and SPD501 neurons, which link CL reduction to the energy-sensitive nigrostriatal pathway [89,113]. Beyond quantity, CL quality changes with disease progression: aged parkin-knockout brains show remodeling toward less unsaturated species and away from highly polyunsaturated forms, a pattern consistent with impaired CL development that reflects ongoing mitochondrial damage [124].

CL topology also shifts early in synucleinopathy. In SNCA-mutant human dopaminergic neurons and A53T transgenic mouse substantia nigra, CL moves to the outer mitochondrial membrane before noticeable fragmentation. This imaging signal indicates that CL profiling can detect changes before structural damage occurs [89]. Functionally, membranes rich in CL influence α-synuclein structure: CL vesicles can shift β-sheet-rich oligomers toward α-helical forms, with slower kinetics observed in A53T/E46K mutants compared to wild-type, implying a genotype-specific buffering ability that CL assays can identify [89,125]. Complementary biophysical studies reveal that CL impacts oligomer size and structure (pH-dependent) and, in models prone to amyloid formation, can accelerate fibril formation and induce moderate toxicity, emphasizing CL’s role at the nexus of aggregation, membrane stress, and ROS [82,112].

Additionally, studies on human tissue emphasize the heterogeneity in cohorts that CL profiling can help stratify. In the substantia nigra of male PD patients, a series of phospholipids decreases while CL mass stays constant; interestingly, these changes are not observed in females. This highlights that measurements of CL (such as content, remodeling, or exposure) may differ depending on sex and disease state [126].

### 3.4. ALS

A growing body of evidence implicates impaired energy metabolism in ALS patients and models [127]. In motor neurons, ALS-associated phenotypes frequently include elevated oxidative stress, protein aggregation, and mitochondrial structural abnormalities [128,129,130]. For example, early studies in presymptomatic mutant SOD1 mice reported degenerating mitochondrial vacuoles in axons and dendrites [131,132,133]. Mitochondrial fragmentation has also been observed in cultured NSC34 cells overexpressing mutant SOD1 [129,134]. Subsequent work further described mitochondrial vacuolation in spinal motor neurons, often discussed in relation to the expansion of the intermembrane space and/or outer mitochondrial membrane, together with altered mitochondrial distribution and disturbed dynamics in the cell body and proximal axon hillock [128,135,136,137,138,139].

Beyond morphology, functional deficits have been documented across ALS settings, including altered mitochondrial electron transport chain activity [140,141,142,143,144], changes in mitochondrial membrane potential, impaired respiratory chain function, and reduced mitochondrial Ca^2+^ buffering capacity [145,146,147]. These mitochondrial impairments are consistent with the notion that neurons (particularly those motor neurons with long axons and high energy demand) are vulnerable to disruptions in aerobic ATP production [106,107].

Since mitochondrial performance depends critically on CL, alterations in CL composition may directly reflect neuronal energy status, making it a valuable indicator of mitochondrial and neuronal function in ALS and related CNS disorders. Research using LC-MS-based lipidomics revealed that ALS progression companies with substantial mitochondrial lipid remodeling, including total CL reduction (by more than 25%), along with specific species decreases (e.g., CL 18:1/18:1/18:1/18:1, CL 16:0/18:1/18:2/18:3, CL 16:0/18:1/20:4/22:6, CL 16:0/18:1/18:1/22:6, CL 16:0/18:1/20:4/18:2, CL 18:1/18:1/20:4/22:6, CL 16:0/18:1/18:1/18:2). Particularly, the species containing C22:6 n-3 and C20:4 n-6 are largely loss half, such as CL 18:1/18:1/22:6/22:6, CL 16:0/18:1/18:2/22:6, CL 16:0/18:1/22:6/22:6, and so on [105,106,107].

It is worth noting that mitochondrial abnormalities in ALS are not restricted to motor neurons. In skeletal muscle from patients, mitochondria are also structurally [148,149] and functionally [150,151] abnormal. Yet, the specific mechanisms require further clarification.

### 3.5. Epilepsy

Epilepsy manifests heterogeneously, ranging from brief lapses in consciousness to convulsive episodes and other neurological impairments [152,153]. ATP production insufficiency caused by malfunctioning mitochondria in brain cells leads to pathophysiological changes in neuronal bioenergetics and metabolism, which frequently result in neurological complications, including recurrent spontaneous seizures, a hallmark of the epilepsies [154,155,156]. Although the underlying mechanisms of epileptogenesis are not well understood, evidence for the involvement of mitochondrial dysfunction is substantial [154,157,158,159,160,161,162,163,164,165,166,167]. At the same time, changes in biomembrane structure have also been observed in some epileptic models as well [168,169].

Johnson et al. utilized FT-ICR/MS to explore the changes in the lipidome in hippocampal and cortical tissue from the Kv1.1-KO model of epilepsy. Their study exhibited significant upregulation of multiple glycerophospholipids (including CL) in the hippocampus compared to the cortex [114]. For the explanation of the results, the authors proposed that seizures generate high oxidative stress, which may preferentially oxidize and reduce CL in the cortex, thereby increasing the hippocampus/cortex ratio; alternatively, metabolic stress during seizures may drive region-specific upregulation of CL synthesis as a compensatory response to stabilize mitochondrial membrane proteins, sustain ATP production, and limit permeability transition pore opening;. At the same time, the most likely scenario involves a combination of both oxidative loss in the cortex and compensatory CL production in the hippocampus [114].

In addition, patients with the most frequent seizures were more likely to have IgM-CL antibodies. The risk for positive IgM-CL, IgG-CL, and β(2)-Gp I antibodies increased concomitantly with increasing intellectual disability [170]. Furthermore, many of the commonly used drugs, including antiseizure drugs (ASDs), can lead to detrimental effects on mitochondrial function and thus are not recommended in patients with confirmed mitochondrial diseases [171].

### 3.6. TBI

TBI rapidly increases neuronal energy demand, driving mitochondria to work at higher respiratory rates, which in turn elevates ROS production and contributes to secondary injury [172,173,174,175]. One key event in this process is the peroxidation of CL, as oxidized CL is known to induce the mitochondrial permeability transition pore, when oxidized CL and its degradation products (e.g., MLCL) accumulate and cannot be purged efficiently, mitochondrial damage worsens, and neurons undergo apoptosis [14,32,176]. Spatial lipid mapping via MALDI-MS imaging further reveals early and region-specific loss of CL (especially those with PUFA) after controlled cortical impact. In the contusional cortex, the ipsilateral hippocampus, and the thalamus, even though there are no visible histological lesions, the mitochondrial membrane is highly vulnerable soon after injury, in which CL of C70 to C76 decreases by approximately 50% compared to the contralateral (less-affected) side/hemisphere [108]. During this worsening phase, CL also translocates from the IMM to OMM, and levels of mitochondrial marker proteins (COXIV, TOM40, and MnSOD) decline, suggesting ongoing mitochondrial loss and dysfunction. All these changes express a clear progression from metabolic stress to mitochondrial failure and neuronal death in TBI [40,108].

In response to this damage, neurons activate mitophagy, and CL plays a central role in signaling. When CL is externalized to the OMM, it serves as a selective “eat-me” signal by binding LC3 to recruit the autophagic machinery [30,32]. The externalized CL mediates targeted LC3-mediated autophagy of damaged mitochondria, which is controlled by PLS3 [177]. Using TEM, an increased amount of autophagic vacuoles containing damaged mitochondria was observed in TBI patients [178]. These findings provide a molecular basis for further exploration of CL as a potential biomarker in TBI.

The spatial pattern of this considerable CL loss is not uniform; still, it reflects a gradient of primary injury severity. The pronounced CL depletion in ipsilateral versus contralateral regions likely reflects the focal severity of key injury mechanisms. Localized oxidative stress and calcium overload at the primary site directly drive CL peroxidation and activate phospholipases, leading to acute, selective hydrolysis. This core damage is further amplified by a spatially constrained neuroinflammatory response, creating a gradient of mitochondrial insult that manifests as the observed differential CL loss.

### 3.7. Spinal Cord Injury (SCI)

Molecular alterations of CL in the context of SCI are identified, including significant reductions in total and polyunsaturated CL species [179,180,181], increased lysoCL formation, and elevated markers of CL oxidation [180]. In differentiating spinal cord motor neuron-like NSC-34 cells, LC–MS/MS reveals a shift away from 18:2 n-6-rich CL species (i.e., CL (18:2 n-6)_4_ and related species) to 18:1 n-9 and 16:0-rich species [181]. Similarly, in the rat spinal cord following contusive SCI, the major unsaturated CL acyl chains, including 18:1 (49.8% of total CL), 20:4 (20.0%), 22:6 (12.3%), and 18:2 (8.1%), were all markedly decreased. Concurrently, lysoCL and the lysoCL/CL ratio were significantly increased, alongside elevated 4-HNE levels indicative of CL oxidation [180].

## 4. Modulation of Cardiolipin by Nutritional Interventions

**Figure 4:**
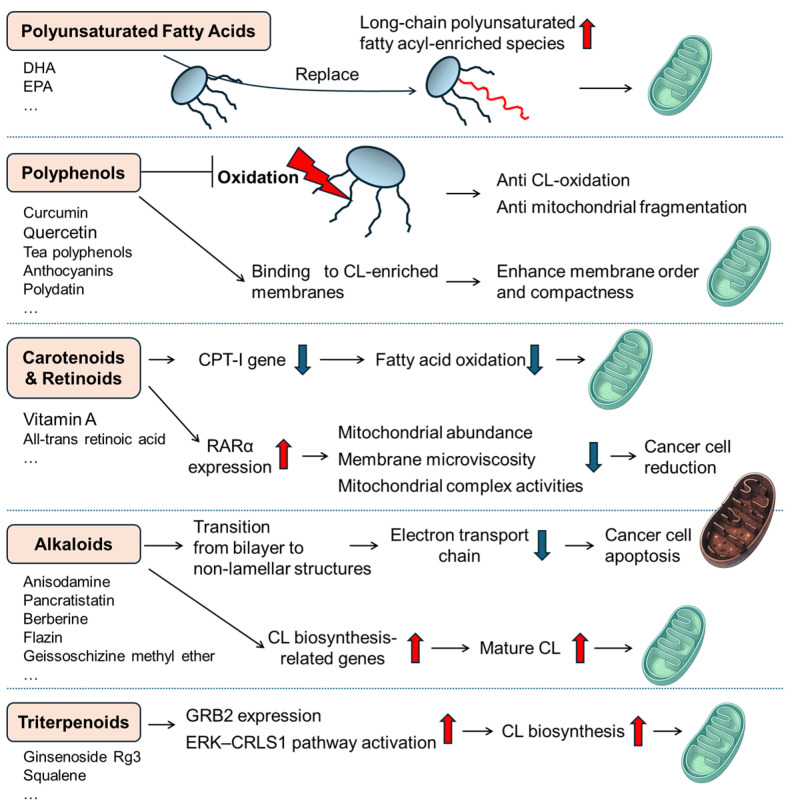
Natural functional components as nutritional interventions that regulate CL and mitochondria through different pathways.

Since CL is essential for maintaining mitochondrial structure and function, which impacts human health and diseases (including CNS disorders, as discussed above), it is crucial to maintain the balance of CL content and its molecular species. Emerging research suggests that various nutritional bioactive components, including fatty acids, polyphenols, alkaloids, carotenoids, and triterpenoids, can positively impact CL remodeling and function (Figure 4). Generally, researchers have been discovering interventions that directly engage with CL at the molecular level, together with remodeling enzymes or membrane physicochemical properties, leading to characteristic shifts in species profiles, such as increased incorporation of long-chain PUFAs, the preservation of mature species (typically, TLCL), and the restoration of the damaged CL molecules (e.g., ox-CL). Crucially, beyond these qualitative changes, key interventions demonstrate the capacity to modulate total CL content, particularly under pathological or deficient conditions. These findings suggest that specific nutraceuticals and functional foods can improve the restoration of cardiolipin and further benefit mitochondrial health, not only for the nervous system but also for the whole body. In this section, we synthesize current evidence on how distinct classes of natural nutritional ingredients modulate CL and maintain human health.

### 4.1. Polyunsaturated Fatty Acids (PUFAs)

Currently, increasing studies have demonstrated that dietary functional fatty acids, particularly n-3 PUFAs such as EPA and DHA, significantly influence mitochondrial CL composition in various tissues and cell models [182]. PUFAs consistently modify the acyl chain composition of CL, usually resulting in higher incorporation of longer-chain and higher-unsaturated fatty acyls, elevation of total CL content in certain situations, and changes in the ratios of representative molecular species, for example, CL (22:6 n-3)_1_(18:2 n-6)_3_, rather than the dominant CL (18:2 n-6)_4_ or CL (20:4 n-6)_1_(18:2 n-6)_3_ [182,183,184,185,186,187]. Berger et al. investigated cardiolipin (CL) levels in the heart, kidney, liver, and spleen of mice given diets supplemented with either 18:3 n-3 or 20:3 n-3. They discovered that dietary 20:3 n-3 is uniquely and extensively incorporated into the CL pool across all organs. In contrast, C18:3 n-3 has minimal direct participation in CL biosynthesis; it is first converted to 22:6 n-3 through elongation and desaturation before incorporation. Notably, CL shows distinct enrichment patterns for these fatty acids, unlike other phospholipids [188]. These results emphasize how dietary n-3 fatty acids significantly influence cardiolipin remodeling in a highly selective, organ-dependent manner, likely by changing acyl chain composition, and suggest potential nutritional strategies for modulating mitochondrial function. Similarly, 20:4 n-6 increased long-chain, more unsaturated CL species and decreased shorter-chain ones. Meanwhile, 20:5 n-3 supplementation significantly raised very-long-chain, highly unsaturated CL species like CL 72:8, CL 74:8, and CL 75:11. Conversely, 22:6 n-3 mainly boosted shorter-chain, less unsaturated species such as CL 68:2, CL 70:3, CL 70:2, and CL 70:4. This comprehensive study demonstrated that different PUFAs selectively influence CL remodeling in mitochondria, leading to alterations in both CL and MLCL profiles [185].

Regarding nutritional supplements, fish oil rich in n-3 PUFAs is shown to specifically modify cardiac CL [184,186], increasing 22:6 n-3 incorporation while reducing 20:4 n-6-containing species, and modulating the balance of CL (18:2 n-6)_4_. This remodeling correlates with a delay in Ca^2+^-induced mitochondrial permeability transition pore opening in healthy hearts but not in infarcted hearts, indicating potential effects on mitochondrial stability [182]. Additionally, using an NAO fluorescence assay to measure CL in primary rat cortical astrocytes, 20:5 n-3 was found to increase mitochondrial cardiolipin levels more significantly than 22:6 n-3, suggesting a stronger ability to alter mitochondrial membranes [187]. However, it is worth noting that Hong et al. demonstrated that dietary n-3 PUFAs from fish oil significantly remodel mitochondrial CL by incorporating 20:5 n-3 and 22:6 n-3 at the expense of 18:2 n-6, but this replacement can increase mitochondrial membrane susceptibility to ROS and predispose colonocytes to apoptosis [184].

### 4.2. Polyphenols

Polyphenols have attracted increasing attention for their antioxidant capacity and modulatory effects on mitochondrial membranes [189,190,191]. Representative compounds such as curcumin and quercetin have been shown to bind preferentially to CL-rich membrane domains, thereby enhancing membrane order and compactness, particularly under oxidative stress conditions [192,193,194]. Natural polyphenols (e.g., those found in tea and certain vegetables) are widely reported to effectively prevent or reverse CL oxidation, contributing to the preservation of mitochondrial membrane integrity and bioenergetic capacity [195,196,197,198]. Moreover, according to Kelsey et al., anthocyanins preserve mitochondrial GSH, inhibit cardiolipin oxidation, suppress mitochondrial oxidative stress-induced apoptosis, and mitochondrial fragmentation [197]. Notably, curcumin is shown to bind to the polar/apolar interface of the lipid bilayers, with its binding favored by CL, which enhances its incorporation into model membranes [193]. Additionally, curcumin restructures cardiolipin-rich membranes by reducing free volume and increasing chain order [192]. Similarly, quercetin can effectively prevent cytochrome c-induced membrane permeabilization by CL oxidation, implying a protective role in early mitochondrial oxidative stress events [194]. Regarding mitochondrial function, Reyna-Bolaños et al. reported that polydatin prevented iron-induced mitochondrial damage under in vitro conditions by preventing increased ROS. As Fe^2+^ impaired the activities of electron transport chain complexes I to IV, polydatin treatment significantly restored the activity of all complexes, with complex I and III activities elevated up to 2-fold above control levels [195].

### 4.3. Carotenoids and Retinoids

Studies have shown that both carotenoids and retinoids are essential for CL composition along with associated bioenergetic processes. They influence CL remodeling primarily through indirect regulatory pathways, including modulation of lipid metabolism, oxidative stress responses, and gene expression. Vitamin A deficiency consistently leads to significant CL depletion in hepatic, cardiac, and testicular mitochondria, accompanied by impaired lipid unsaturation and altered phospholipid ratios [199,200,201,202]. An in vivo study found that feeding the vitamin A-deficient diet to rats increased CPT-I activity and gene expression, leading to a significant rise in mitochondrial fatty acid oxidation and a notable decrease in cardiolipin content (*p* < 0.01) [199]. Consistently, in another study of heart (ventricular) lipids, vitamin A deficiency induced mitochondrial CL decrease; at the same time, heart activity and mRNA levels of CPT-I and expression of PPAR-α and PPAR-β genes were enhanced, whereas acetyl-coenzyme A carboxylase activity diminished [200].

Terao et al. used LC-MS lipidomics to investigate the impact of all-trans retinoic acid (ATRA) on CL profiles in breast cancer cells with different RARα expression. In ATRA-sensitive SK-BR-3 cells, total CL levels were reduced, notably CL 72:8 and CL 70:6. RARα-overexpressing cells also showed significant CL downregulation; in contrast, RARα-knockdown cells exhibited unchanged or increased CL levels. These changes correlated with decreased mitochondrial abundance, membrane microviscosity, and suppressed mitochondrial complex activities, indicating that ATRA remodels mitochondrial CL composition in a RARα-dependent manner, leading to mitochondrial dysfunction and antiproliferative effects [202]. The reduction effect of ATRA on the overall CL amount in multiple cell lines (SK-BR-3, HCC-1500, CAMA1, and MDA-MB-361) suggests its potential as an anticancer agent [202].

### 4.4. Alkaloids

Some alkaloids are known to directly interact with lipid membranes, especially CL-enriched domains, thereby modulating mitochondrial structure and function. For example, anisodamine directly changes the physical structure of CL-rich membranes. In CL liposomes, adding approximately 30 mol% anisodamine causes a transition from lamellar to non-lamellar phases, forming around 10 nm lipidic particles at pH 7.0 and H_II hexagonal nanotubes (~7.3 nm) at pH 8.8. This phase change results from increased disorder in acyl chains (gauche conformers) and altered headgroup packing due to electrostatic interactions, indicating its ability to make CL-containing membranes more sensitive to curvature stress, which is vital for mitochondrial cristae remodeling and apoptotic susceptibility [203]. Similarly, pancratistatin, a natural antiviral and anticancer alkaloid with strong mitochondrial selectivity, promotes the formation of non-lamellar phases in CL membranes. The H_II structures emerge as low as 10 μM pancratistatin in pure CL liposomes, accompanied by disrupted lipid ordering and distribution within IMM mimetic systems. Therefore, pancratistatin-induced CL reorganization is linked to impaired maintenance of IMM protein function, contributing to selective activation of mitochondrial apoptotic signaling in cancer cells while sparing non-malignant cells [204].

Besides disrupting CL homeostasis, certain alkaloids are found to restore CL content and species composition under metabolic stress. Cole et al. found that in mice with gestational diabetes induced by a high-fat diet, cardiac CL levels were decreased but notably restored through dietary berberine supplementation. The mechanism involved berberine upregulating tafazzin mRNA expression and restoring the intact functional CL species, which was associated with enhanced mitochondrial respiration and improved cardiac metabolic function [205]. Geissoschizine methyl ether, an indole alkaloid from Uncaria hook, protects CL metabolism under neurotoxic oxidative stress. In glutamate-challenged neuronal models, geissoschizine methyl ether reversed the suppression of CL-related genes (Cds1, Crsl1, Pla2g6) and further upregulated Tamm41, Taz, and Lclat1. This gene-level regulation preserves CL content and mitochondrial membrane integrity, preventing excitotoxicity-related mitochondrial dysfunction [206]. In addition, a food-derived β-carboline alkaloid, flazin, was found to improve preserved mitochondrial morphology in renal tubular epithelial cells under oxidative stress. Importantly, flazin significantly increased total CL content, reduced CL hydroperoxides, and improved the CL profile, replacing short-chain CL species with long-chain species; therefore, it is considered a nutraceutical candidate that contributes to the treatment of oxidative stress-induced mitochondrial disturbance [54].

### 4.5. Triterpenoids

Triterpenoids have the potential to contribute to the restoration of mitochondrial membrane integrity and function in various disease contexts [207,208]. Ginsenoside Rg3 significantly restored rotenone-induced reduction in brain CL and upregulated GRB2, which further activated the ERK-CRLS1 signaling pathway, increasing CL biosynthesis via mitochondrial CL synthase. These results suggest that ginsenoside Rg3 modulates CL remodeling and restores mitochondrial membrane integrity, thereby contributing to neuroprotection in PD [90]. Herrera-Marcos et al. investigated dietary squalene (0.5% *w*/*w*; ~135 mg/kg/day) in a porcine NASH model and observed a selective reduction in the cardiolipin species CL 69:5, decreasing from 3.5 to 0.4 pmol/mg liver tissue (*p* < 0.001), with no significant change in total CL. Interestingly, no other CL species was significantly affected. CL 69:5 showed strong negative associations with hepatic squalene levels and acted as a lipid network hub linked to multiple lipid classes, suggesting species-specific remodeling rather than global CL depletion [207]. Furthermore, the combination of triterpenoids and alkaloids exhibited a CL-regulating effect. Wang et al. evaluated serum CL levels in a rat model of chronic heart failure induced by transverse aortic constriction, and oral treatment with a combination of hypaconitine and glycyrrhetinic acid resulted in a significant upregulation of CL levels [208].

### 4.6. Prebiotics and Short-Chain Fatty Acids (SCFAs)

SCFAs, mainly generated through gut microbial fermentation of dietary fibers, have become important mediators connecting host metabolism, immune signaling, and mitochondrial function [209,210,211]. Meanwhile, prebiotics like inulin, which enhance SCFA production and impact gut microbiota, could indirectly alter mitochondrial lipid composition, especially the polyunsaturated species. These changes might weaken mitochondrial membrane integrity and impair function [212]. On the other hand, however, Sun et al. found that inulin could regulate the pathways of fatty acid biosynthesis and CL synthesis, promoting β-oxidation level, preventing lipogenesis, suppressing lipid accumulation and oxidation in hepatocytes of non-alcoholic fatty liver disease (NAFLD)-modeled mice [213].

## 5. Conclusions

Significant progress has been achieved in developing CL measurement and applying it to biological studies, as well as understanding its modulation by nutritional supplementation. Nevertheless, limitations in both analysis and nutrition intervention remain to be further addressed. One critical concern lies in clinical translation. While considerable investigation has been conducted on pathology-related CL alterations in animal models and cell lines, studies on humans (especially human tissues) are still relatively limited. Most approaches are highly invasive (such as brain biopsies), requiring handling techniques while bringing pain to subjects, thus not practical for routine diagnosis or monitoring. Moreover, although blood sampling is accessible, it is much less abundant in CL compared with tissues and lacks organ/tissue/region specificity. Therefore, it is of great need to develop sensitive and non-invasive methods for profiling CL along with its related metabolic products to enable full-stage clinical monitoring of mitochondrial health in not only CNS diseases but also other disorders.

Furthermore, the field also faces a lack of standardized methodologies. Variations in extraction protocols, platforms (for example, even for the LC-MS approach, there are many different instrumentations), and data normalization methods make it difficult to compare studies directly and to set universal reference ranges or diagnostic thresholds. Thus, the quantitative interpretation of CL changes across studies remains difficult, which affects its widespread application in the clinic. A more challenging point is the absence of tools to monitor CL remodeling and oxidation in vivo effectively: most available data are static snapshots from post-mortem tissues or biofluids, which cannot capture the real-time flux of CL metabolism, which should be a key factor for understanding the initial pathogenic processes.

Another major limitation is the indirect regulation of CL modulation. As discussed above, almost all nutritional supplements influence CL biosynthesis and remodeling through systemic metabolic and/or specific signaling pathways. In contrast, strategies that directly aim at the mitochondrial membrane might be of interest in the future, which could enable the supplementation of CL (or its precursors) specifically targeting IMM, for instance, by delivering mature CL species, replacing damaged molecules, and restoring membrane integrity.

Therefore, advancing our knowledge of CL in CNS disorders will, no doubt, increasingly depend on the integrative analysis of CL data within a multi-omics framework. Correlating CL lipidomics with transcriptomics, proteomics, and metabolomics will build more comprehensive network models of mitochondrial dysfunction. For instance, using LC-HRMS-based lipidomics for discovery, MS imaging for spatial validation, and biophysical tools (e.g., AFM-IR) combined with additional functional assays (e.g., Seahorse analysis) to establish direct mechanistic links between CL chemistry and organelle function.

In conclusion, explorations integrating CL analysis on clinical and nutritional applications enhance our understanding of mitochondria-related disorders, especially CNS diseases. Conducting more in-depth research can help improve mitochondrial health and promote overall human well-being.

## Figures and Tables

**Table 1:** Comparison of different CL analysis approaches.

Approach	Aims in CL Research	Key Features
LC-MS	Composition/Remodeling (CL and lysoCL profiling); Oxidation (ox-CL species)	Provides high-resolution separation of CL species before MS analysis. Ideal for detailed lipid profiling, identification of oxidized CL, and relative quantification in complex biological extracts.
Shotgun MS	High-throughput CL profiling	Enables direct infusion MS analysis without prior chromatography. Offers rapid, broad-coverage screening of CL and other lipids, suitable for large sample sets and biomarker discovery.
MS Imaging	Spatial mapping (tissue regions, cellular distribution, etc.)	Allows label-free, in situ visualization of CL distribution directly from tissue sections. Can be coupled with tandem MS for spatial mapping of specific CL molecular species.
NMR	Mechanism; Interactions (CL-protein, membrane interactions)	Delivers atomic-resolution insights into CL structure, dynamics, and molecular interactions in solution. The method of choice for mechanistic studies of binding and conformational changes.
FTIR	Chemical fingerprinting; bulk phase behavior and lipid order	Probes vibrational modes of functional groups, providing information on CL acyl chain order, phase state, and general chemical composition in bulk or model membrane samples.
AFM-IR	Nanoscale spatial mapping; biophysical properties (phase/order, chemistry)	Combines nanoscale topographic imaging with chemical identification via IR. Uniquely capable of correlating CL domain morphology with chemical composition at sub-micron resolution.
Fluorescent Probe	Live-cell dynamics (redistribution, process tracking, etc.)	Enables real-time, non-invasive visualization of CL localization and mitochondrial dynamics in living cells using specific dyes or genetically encoded sensors.

## Data Availability

No new data were created or analyzed in this study. Data sharing is not applicable to this article.

## Abbreviations

The following abbreviations are used in this manuscript:

AD	Alzheimer’s disease
AFM	Atomic force microscopy
ALS	Amyotrophic lateral sclerosis
ATRA	All-trans retinoic acid
Aβ	Amyloid-β
Cer	Ceramide
CID	Collision-induced dissociation
CL	Cardiolipin
CNS	Central nervous system
DESI	Desorption electrospray ionization
DHA	Docosahexaenoic acid
ER	Endoplasmic reticulum
ESI	Electrospray ionization
FTIR	Fourier-transformed infrared spectroscopy
HILIC	Hydrophilic interaction liquid chromatography
HR-MS	High-resolution mass spectrometry
IMM	Inner mitochondrial membrane
MALDI	Matrix-assisted laser desorption ionization
MLCL	Monolysocardiolipin
MPTP	Mitochondrial permeability transition pore
MS	Mass spectrometry
NAFLD	Nonalcoholic fatty liver disease
NAO	Nonyl acridine orange
NMR	Nuclear Magnetic Resonance
OMM	Outer mitochondrial membrane
PA	Phosphatidic acid
PD	Parkinson’s disease
PG	Phosphatidylglycerol
PUFA	Polyunsaturated fatty acid
ROS	Reactive oxygen species
RPLC	Reverse-phase liquid chromatography
SCFA	Short-chain fatty acid
SM	Sphingomyelin
TBI	Traumatic brain injury
TEM	Transmission electron microscopy
TLCL	Tetralinoleoyl CL
UPLC	Ultra-performance liquid chromatography
